# The role of immunosuppression in long-term graft hepatitis and fibrosis after paediatric liver transplant – comparison of two treatment protocols

**DOI:** 10.3389/frtra.2022.1042676

**Published:** 2023-02-28

**Authors:** Wolfram Haller, James Hodson, Rachel Brown, Carla Lloyd, Stefan Hubscher, Patrick McKiernan, Deirdre Kelly

**Affiliations:** ^1^ Department of Gastroenterology & Nutrition, Birmingham Woman's and Children's Hospital NHS Foundation Trust, Birmingham, United Kingdom; ^2^ Institute of Clinical Sciences, University of Birmingham, Birmingham, United Kingdom; ^3^ Research Development and Innovation, Institute of Translational Medicine, University Hospitals Birmingham NHS Foundation Trust, Birmingham, United Kingdom; ^4^ Department of Cellular Pathology, Queen Elizabeth Hospital, Birmingham, United Kingdom; ^5^ Liver Unit, Birmingham Woman's and Children's Hospital NHS Foundation Trust, Birmingham, United Kingdom; ^6^ Institute for Immunology and Immunotherapy, University of Birmingham, Birmingham, United Kingdom

**Keywords:** histology, outcome, fibrosis, allograft, rejection, liver biopsy

## Abstract

**Background and aims:**

We have previously demonstrated high rates of chronic allograft hepatitis and fibrosis in liver transplant patients on long-term cyclosporine monotherapy. We subsequently changed practice to add low-dose prednisolone to maintenance treatment with tacrolimus post-transplant. The aim of the study was to assess the impact of the immunosuppression change on graft histopathology.

**Methods:**

Patients treated in this era (Tac + Pred, 2000–2009, *N* = 128) were compared to a historical cohort, who had been maintained on a steroid-free, cyclosporine-based regime (CSA-Only, 1985–1996, *N* = 129). Protocol liver biopsies and laboratory tests were performed five- and ten-years post-transplant in both groups.

**Results:**

Compared to CSA-Only, the Tac + Pred cohort had significantly lower rates of chronic hepatitis (CH) at five (20% vs. 44%, *p* < 0.001) and ten (15% vs. 67%, *p* < 0.001) years post-transplant, with similar trends observed in inflammation and fibrosis at five years. The Tac + Pred cohort also had significantly lower hepatic transaminases and IgG levels and was less likely to be autoantibody positive at both time points. However, the degree of graft fibrosis at ten years did not differ significantly between eras (*p* = 0.356).

**Conclusion:**

Increased immunosuppression effectively reduced chronic allograft hepatitis and fibrosis at five years, suggesting it is an immunologically driven variant of rejection. However, there was no significant reduction in the degree of fibrosis at ten years, indicating a multifactorial origin for long term graft fibrosis.

## Introduction

Liver transplantation is an established life-saving treatment for children with acute and chronic liver failure. Advances in surgical, anaesthetic and medical management have substantially reduced early post-transplant mortality, with long-term survival rates of patients transplanted for chronic liver disease consistently exceeding 80% (1, 2). Therefore, the emphasis of treatment protocols has shifted from short-term survival, avoidance of surgical complications, and cellular rejection, to long-term allograft function and recipient quality of life. Minimisation of immunosuppression (IS)-related morbidity is one of the central treatment goals. Identifying ways to safely minimise exposure to toxic agents, such as corticosteroids and calcineurin inhibitors, as well as recognition of those tolerant to near or complete IS withdrawal are key outcome targets. This is particularly relevant in the paediatric transplant cohort, where one graft ideally lasts a whole lifespan.

Whilst there is agreement on the need to personalize IS strategies (3–5), determining the minimum degree of IS to protect the graft from immunological damage is challenging. Detection of allograft damage using routine biochemical parameters, such as transaminases, is ineffective (6, 7). In contrast, protocol biopsies have an important role in long-term surveillance of allograft health (8–10) and in guiding IS manipulation (8).

We and others have previously reported unexplained chronic hepatitis (CH), described as idiopathic post-transplant hepatitis (IPTH) by the Banff Working Group in 2006 (11), as a common phenomenon after paediatric liver transplantation, and an important contributor to progressive allograft fibrosis (6, 12). At that time, our protocol was based on CNI monotherapy with steroid withdrawal by three months post-transplantation. Whilst the aetiology and pathogenesis are incompletely understood, there is increasing evidence to suggest that late graft inflammation and fibrosis in paediatric liver allograft recipients are related to alloimmune injury, which may involve both *T*-cell and antibody mediated mechanisms (3, 13, 14). Consequently, we changed our immunosuppressive protocol in 2000 to continue long-term low-dose maintenance steroid treatment for all paediatric allograft recipients.

The main aim of this study was to compare the histological outcomes between two treatment eras. Other study aims included comparing serum biochemical and immunological markers, rates of rejection, biliary and vascular complications and patient growth between the two groups, and investigating possible factors associated with the development of graft fibrosis.

## Materials and methods

### Study subjects

Children (aged <16 years) under the care of Birmingham Children's Hospital receiving a first isolated liver transplantation were retrospectively identified from a departmental database. Patients transplanted for IFALD (intestinal failure associated liver disease), and those receiving a combined graft were excluded. The primary outcomes were based on five- and ten-year protocol biopsies. Hence, patients that died, were re-transplanted after more than seven days, did not have a five-year protocol review, or were lost to follow-up prior to five years were excluded. Early re-transplantation (within seven days) was treated as the index transplant.

Data were extracted for patients transplanted during two eras, defined by the IS maintenance treatment used in the department at the time. The first era included a historical cohort of patients transplanted between 1st January 1985 and 31st December 1996, which has previously been reported by Evans et al. (6) During this period, the first-line IS was cyclosporine A (CSA). Prednisolone and Azathioprine treatment were commenced immediately post-transplant but were subsequently discontinued at three months and 12 months post-transplantation, respectively. Hence, this era is referred to as “CSA-Only”.

The second era included patients transplanted between 1st January 2000 and 31st October 2009. During this period, post-transplant IS included induction with an anti-IL2 receptor agent (daclizumab until 2004, and basiliximab subsequently), corticosteroids and tacrolimus. During the early period of the study, there was a small number of patients who had Prednisolone added to CSA as their first-line IS. From three months post-transplant, all patients were managed with a calcineurin inhibitor along with long-term, low-dose corticosteroid maintenance (target: prednisolone 0.1 mg/kg od). This era is referred to as “Tac + Pred” subsequently.

A number of patients in both eras changed their first line CNI-based IS to Azathioprine or MMF for reasons of nephrotoxicity or histology-proven CH; these were treated as being on “Other” first-line IS for analysis.

Target levels were between 60 and 90 µg/L for cyclosporine, and between 3 and 5 µg/L for tacrolimus. Renal dysfunction was managed by CNI dose reduction or withdrawal, with concomitant introduction of mycophenolate or Azathioprine as a renal sparing agent. Azathioprine was also introduced in patients who displayed features of CH on protocol (please refer to definition below, “histological criteria”) or *ad hoc* biopsies (performed *ad hoc* when concerns about rejection). Sirolimus was used in patients with chronic rejection or medication non-adherence.

The CNI, or IS replacing this, was referred to as the “first-line” IS, whilst additional IS drugs added alongside the first-line treatment were referred to as “second-line” IS.

Consent for anonymized data usage was granted by our Governance Service Unit (clinical audit number CARMS-00201).

### Patient follow-up

Baseline patient- and transplant-related characteristics were documented (diagnosis at transplant, gender, CMV serostatus, graft type, cold ischaemic time [CIT] and blood group mismatch). Blood group mismatch was defined as “major” for AB0 incompatibility, or as “minor” for 0 to A, B, AB incompatibility. All patients were initially followed at the paediatric liver unit, with transition to adult care when appropriate. During this time, any episodes of rejection, or biliary and vascular complications were documented. In addition, a protocol review was performed after both five and ten years of follow-up, which consisted of the following:

• Clinical review with documentation of current IS.
• Percutaneous liver biopsy.
• Blood sampling within 48 h of liver biopsy, to assess standard liver function tests (including bilirubin, albumin, ALT, AST, GGT and ALP), immunoglobulins (IgG, IgM, and IgA) and autoantibodies (AABs: ANA, SMA and LKM) which were classified as positive when ≥1:80.

### Protocol biopsy and histological criteria

Liver sections were stained with haematoxylin and eosin, haematoxylin van Gieson, reticulin, orcein, Perls, and periodic acid-Schiff, with and without diastase pre-treatment. All liver biopsies were assessed by the same histopathologist (R.M.B.), who was blinded to the clinical history. Histological findings were allocated to six diagnostic categories, comparable to the classification used in our original report (6). These consisted of: normal/near normal; chronic hepatitis; rejection (acute *T*-cell mediated rejection and chronic rejection); biliary obstruction; recurrent disease; and other histopathological abnormalities. Near normal changes in the previous study were defined as mild inflammation (portal and/or lobular) without interface involvement or confluent necrosis and/or mild (stage 1) fibrosis. In assessing biopsies for the present study, we noticed that there were some cases in which more than mild fibrosis occurred in combination with inflammation minimal enough to be classified as “near normal” (isolated fibrosis). In order to make comparisons with the historical cohort, cases with isolated fibrosis were included in the (near) normal cohort. To make statistical analysis more meaningful, we combined cases of rejection, biliary obstruction, and recurrent disease with those classified as “other histopathological abnormalities” to form a single “other” category.

CH was defined as a mononuclear portal infiltrate, with variable degrees of interface activity and/or lobular inflammation associated with hepatocyte necrosis, and without features of acute or chronic rejection, or any other identifiable causes of graft injury. Other publications have used the term “idiopathic post-transplant hepatitis”(11, 15) to describe a comparable histologic phenotype. However, we continue to use “chronic hepatitis”, for compatibility with the study by Evans et al. in 2006.

For the purpose of comparability of the two eras, the semi-quantitative histological assessment score previously used by Evans et al. (6) has been used in this study as well. Parenchymal inflammation was divided into lobular and interface-type. Each was scored semi-quantitatively on a four-point scale of 0 to 3 (0 = none, 1 = mild, 2 = moderate, 3 = severe). Interface and lobular inflammation scores were then combined in an overall grade of inflammatory activity. Staging of fibrosis was carried out in a similar way on a four-point scale (0 = none, 1 = mild periportal/pericentral without bridging, 2 = moderate with bridging fibrosis, 3 = severe [i.e., cirrhosis]).

### Statistical analysis

Initially, patient characteristics and outcomes were compared between the two treatment eras. Normally distributed variables were reported as mean ± standard deviation (SD) and compared between groups using independent samples *t*-tests. Non-normal variables were reported as median (interquartile range, IQR), and analysed using Mann-Whitney *U* tests. Ordinal variables were also compared using Mann-Whitney *U* tests, whilst nominal variables were analysed using Fisher's exact tests where these were calculable, or Chi-square tests otherwise. For auxological outcomes, z-scores at baseline were compared to a value of zero using a one-sample *t*-test, whilst comparisons between discharge and follow up assessments were analysed using paired t-tests, and comparisons between treatment eras were performed using independent samples *t*-tests.

Associations with inflammation and fibrosis were then assessed. Ordinal and continuous variables were analysed using Spearman's (rho) correlation coefficients, whilst nominal variables were assessed using either Mann-Whitney *U* tests or Kruskal-Wallis tests, for variables with two or more than two categories, respectively. Multivariable analyses were also produced, in order to assess the impact of potentially confounding factors on the comparison of histological findings between treatment eras. Biopsy outcomes were dichotomized and analysed using binary logistic regression models. Due to the sample size, it was not possible to produce reliable models adjusting for all baseline factors; hence, only those that were found to differ significantly between treatment eras were included.

All analyses were performed using IBM SPSS 22 (IBM Corp. Armonk, NY), with *p* < 0.05 deemed to be indicative of statistical significance throughout.

## Results

### Era characteristics and follow up

**Figure 1:**
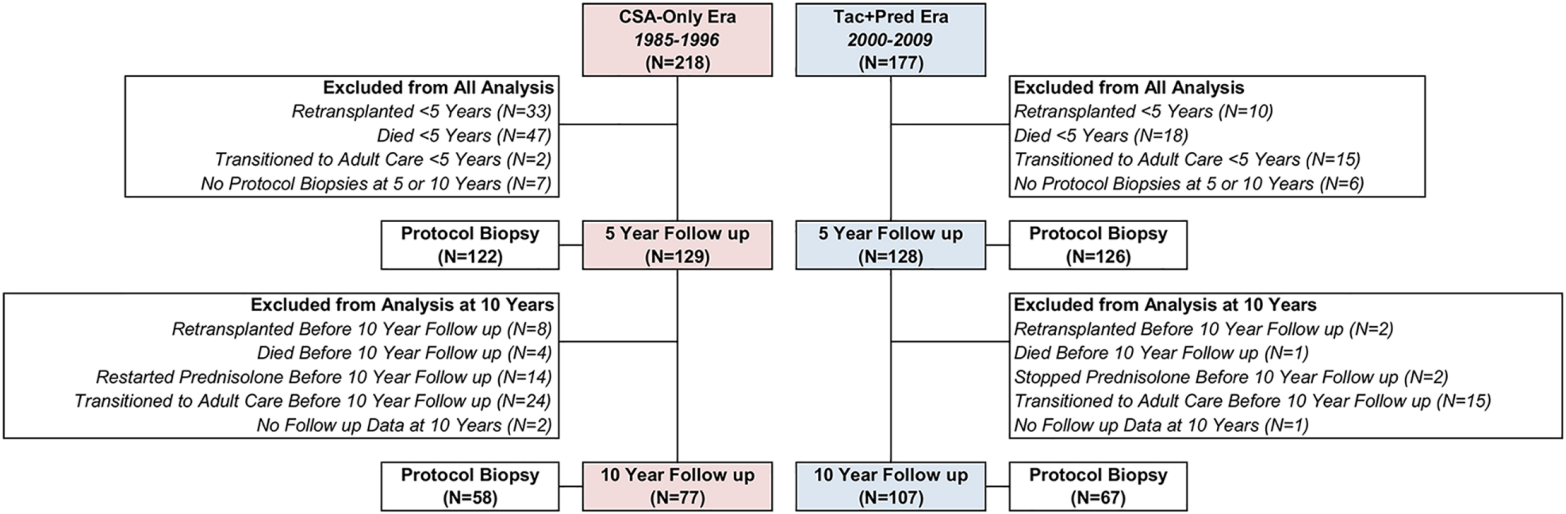
**Selection of study participants for five- and ten-year protocol visits by era.** Follow up at 5 and 10 years was within ±3 years. Reasons for exclusions are mutually exclusive, and are classified in the order listed, i.e., a patient that was retransplanted and then died will be counted as retransplanted.

Details of patient follow up and exclusions are reported in Figure 1. A total of 218 patients transplanted during the CSA-Only era (1985–1996), and 177 transplanted during the Tac + Pred era (2000–2009) were identified. Of these, 108 patients either died or were retransplanted within five years of the index transplant, with this occurring significantly more commonly in the CSA-Only era (37% vs. 16%, *p* < 0.001). A further 17 were transitioned to adult care prior to five years, and subsequently lost to follow up, whilst 13 did not undergo protocol biopsies at either five or ten years.

After these exclusions, data from the five-year (± three years) protocol review were available for 129 patients from the CSA-Only era, and 128 from the Tac + Pred era. The timing of the five-year follow up assessment was similar in both eras, with means of 5.0 ± 0.7 and 5.1 ± 0.5 years from transplant, respectively (*p* = 0.465). Of patients for whom clinical assessments were available, 122 (95%) and 126 (98%) from the CSA-Only and Tac + Pred eras, respectively, underwent protocol biopsies.

After the five-year assessment, a further 15 patients subsequently either died or were retransplanted less than ten years after the index transplant. In addition, 39 were transitioned to adult care, and 3 were lost to follow up before ten years. Within the CSA-Only group, 14 patients restarted prednisolone treatment before ten years, due to a deterioration of symptoms/biopsy findings of chronic hepatitis, whilst 2 from the Tac + Pred era stopped prednisolone; these patients were excluded from analysis of ten-year outcomes, since they deviated from the standard IS regimen of the era. After exclusions, data from the ten-year clinical assessments were available for 77 from the CSA-Only era, and 107 from the Tac + Pred era, which were performed a mean of 9.9 ± 1.1 and 10.1 ± 0.7 years from the index transplant, respectively (*p* = 0.100). Protocol biopsy data were available for 58 (75%) and 67 (63%) of those attending the ten-year assessment within the CSA-Only and Tac + Pred eras, respectively.

### Immunosuppressive regimens

The maintenance IS regimens in the two eras are summarized in Table 1. In the CSA-Only era, all patients received cyclosporine as first-line IS; 98% of patients received azathioprine as a second-line, combination treatment, with planned discontinuation after 12 months post-transplant. In the Tac + Pred era, tacrolimus was the most common first-line treatment at discharge (95%), with the remaining 5% of patients from early in the study period receiving cyclosporine. The majority of patients in this era did not receive a second-line therapy at discharge (78%), with the remainder treated with MMF (17%) or azathioprine (5%). These second-line discharge therapies were used during the later study period to reduce the dose of CNI needed and the associated risk of nephrotoxicity.

**Table 1:** Maintenance immunosuppression regimens by era.

	CSA-Only			Tac + Pred		
	*Discharge*	*Five Years*	*Ten Years*	*Discharge*	*Five Years*	*Ten Years*
**First-line Immunosuppression**						
* Tacrolimus*	0 (0%)	0 (0%)	2 (3%)	121 (95%)	119 (93%)	98 (93%)
* Cyclosporine*	129 (100%)	129 (100%)	54 (77%)	7 (5%)	6 (5%)	2 (2%)
* Sirolimus*	0 (0%)	0 (0%)	0 (0%)	0 (0%)	2 (2%)	4 (4%)
* Other*	0 (0%)	0 (0%)	14 (20%)	0 (0%)	1 (1%)	1 (1%)
**Second-line Immunosuppression**						
* None*	2 (2%)	108 (92%)	42 (67%)	100 (78%)	102 (80%)	77 (72%)
* Azathioprine*	127 (98%)	8 (7%)	6 (10%)	6 (5%)	4 (3%)	8 (7%)
* MMF*	0 (0%)	1 (1%)	15 (24%)	22 (17%)	19 (15%)	20 (19%)
* Sirolimus*	0 (0%)	1 (1%)	0 (0%)	0 (0%)	3 (2%)	2 (2%)

CSA, cyclosporine A; MMF, mycophenolate mofetil, Pred, prednisolone; Tac, tacrolimus.

Whilst the distribution of first- and second-line medications remained similar at five and ten years in the Tac + Pred era, immunosuppression in the CSA-Only era was less consistent. At ten years, 3% of patients had been swapped from CSA to tacrolimus as their first-line IS. For reasons of biopsy-proven CH and nephroprotection, some patients had Azathioprine or MMF introduced as first-line (other = 20%) or second line IS (10% and 24% respectively) at ten years.

### Baseline patient demographics

Baseline cohort characteristics are summarized in Table 2. Patients transplanted in the Tac + Pred era were significantly younger (median: 1.9 vs. 2.9 years, *p* = 0.024) than those in the CSA-Only era. The distribution of diagnoses was similar in the two eras (*p* = 0.276), with biliary atresia (47%) being the predominant reason for transplant. Of the transplant-related factors, the later Tac + Pred era used significantly more split grafts (54% vs. 2%, *p* < 0.001), and organs from older donors (median: 22 vs. 10 years, *p* < 0.001), with a significantly shorter CIT (mean: 558 vs. 666 min, *p* < 0.001) than the CSA-Only era.

**Table 2:** Baseline cohort characteristics by era.

	CSA-Only		Tac + Pred		*p*-Value
	N	*Statistic*	N	*Statistic*	
Age (Years)					
* Recipient*	129	2.9 (0.9–8.4)	128	1.9 (0.8–5.2)	**0**.**024**
* Donor*	129	10.0 (6.0–16.0)	127	22.0 (13.4–34.0)	**<0**.**001**
Gender					
* Recipient (% Male)*	129	64 (50%)	128	55 (43%)	0.318
* Donor (% Male)*	129	77 (60%)	125	85 (68%)	0.192
* Donor/Recipient Match*	129	66 (51%)	125	58 (46%)	0.455
Diagnosis	129		128		0.276**
* Biliary Atresia*		63 (49%)		58 (45%)	
* Metabolic*		20 (16%)		22 (17%)	
* Acute Liver Failure*		16 (12%)		17 (13%)	
* Cholestasis*		16 (12%)		8 (6%)	
* Malignancy*		4 (3%)		6 (5%)	
* Autoimmune Liver Disease*		2 (2%)		5 (4%)	
* Other*		0 (0%)		4 (3%)	
* Indeterminate*		8 (6%)		8 (6%)	
CMV Serostatus					
* Recipient (% Positive)*	128	44 (34%)	126	33 (26%)	0.173
* Donor (% Positive)*	127	46 (36%)	125	47 (38%)	0.896
Blood Group Mismatch	125		125		0.798*
* No*		108 (86%)		107 (86%)	
* Minor*		17 (14%)		15 (12%)	
* Major (ABOi)*		0 (0%)		3 (2%)	
Graft Type	129		128		**<0**.**001**
* Whole*		60 (47%)		18 (14%)	
* Split*		2 (2%)		69 (54%)	
* Reduced*		67 (52%)		41 (32%)	
CIT (Minutes)	129	666 ± 210	114	588 ± 120	**<0**.**001**

Data are reported as N (Column %), with *p*-values from Fisher's exact tests; median (interquartile range), with *p*-values from Mann-Whitney U tests; or as mean ± SD, with *p*-values from independent samples t-tests, unless stated otherwise. Bold *p*-values are significant at *p* < 0.05.

* *p*-Value from Mann-Whitney *U* test, as the factor is ordinal.

** *p*-Value from Chi-square test, as Fisher's exact test was incalculable. ABOi, ABO incompatible; BMI, body mass index; CMV, cytomegalovirus; CIT, cold ischaemic time; CSA, cyclosporine A; Pred, prednisolone; Tac, tacrolimus. Comparisons between the cohorts found the Tac + Pred cohort to have significantly younger recipients, older donors, a higher proportion of split grafts and shorter cold ischaemic times.

Analysis of the subgroup of patients with ten-year protocol biopsies returned similar results (see Supplementary Table A).

### Rejection outcomes and biliary/vascular complications

In the Tac + Pred era, 55% of patients developed at least one episode of acute TCMR, which was similar to the 49% in the CSA-Only era (*p* = 0.379, Table 3). The numbers of rejection episodes were also similar in the two eras, with a mean of 0.5 vs. 0.4 (*p* = 0.158) early and 0.2 vs. 0.2 (*p* = 0.668) late acute TCMR episodes per patient in the Tac + Pred vs. CSA-Only eras. Chronic rejection rates were also similar in the two eras (5% vs. 9%, *p* = 0.221), as were the rates of biliary and vascular complications.

**Table 3:** Rejection outcomes and complications by era.

	CSA-Only		Tac + Pred		*p*-Value
	*N*	*Statistic*	*N*	*Statistic*	
Any Acute TCMR	126	62 (49%)	128	71 (55%)	0.379
Number of Early Acute TCMR Episodes	126		128		0.158*
* 0*		80 (63%)		69 (54%)	
* 1*		37 (29%)		50 (39%)	
* 2*		9 (7%)		8 (6%)	
* 3*		0 (0%)		1 (1%)	
Number of Late Acute TCMR Episodes	126		128		0.668*
* 0*		102 (81%)		106 (83%)	
* 1*		19 (15%)		19 (15%)	
* 2*		4 (3%)		2 (2%)	
* 3*		1 (1%)		1 (1%)	
Chronic Rejection	127	11 (9%)	128	6 (5%)	0.221
Biliary Complication	127	20 (16%)	128	13 (10%)	0.197
Vascular Complication	127	6 (5%)	128	13 (10%)	0.151

Data are reported as N (Column %), with *p*-values from Fisher's exact tests, unless stated otherwise. Bold *p*-values are significant at *p* < 0.05. “Early” was classified as rejection within 90 days, with “late” referring to rejection after 90 days.

* *p*-Value from Mann-Whitney *U* test, as the factor is ordinal. The analysis found no evidence of significant differences between the cohorts with respect to rejection, or biliary or vascular complications. CSA, cyclosporine A; Pred, prednisolone; Tac, tacrolimus; TCMR, T-cell mediated rejection.

### Laboratory findings

At the time of the five year follow up, patients in the Tac + Pred era were significantly less likely to have developed autoantibodies (18% vs. 47%, *p* < 0.001), and had significantly lower ALT (median: 20 vs. 30 IU/L, *p* < 0.001), AST (34 vs. 45 IU/L, *p* < 0.001) and GGT (15 vs. 23 IU/L, *p* < 0.001) levels than those in the CSA-Only era (Table 4). The results were comparable when using ratios of the absolute transaminase level to the age-relevant upper limit of normal, acknowledging different analysis methods used at different era time points. Significantly higher albumin levels (*p* < 0.001) and lower IgG levels (*p* < 0.001) were also observed in the Tac + Pred era at five years, compared to the CSA-Only era. All of these differences persisted at the ten-year follow up. At the five-year follow up, patients in the Tac + Pred era also had significantly lower bilirubin, IgA and IgM, and higher ALP levels than the CSA-Only era, although these differences were no longer significant at the ten year follow up.

**Table 4:** Laboratory findings by era.

	CSA-Only		Tac + Pred		*p*-Value
	*N*	*Statistic*	*N*	*Statistic*	
**Five Year Follow-up**	***N = 129*** *		***N = 128*** *		
Autoantibodies (% Positive)	119	56 (47%)	123	22 (18%)	**<0**.**001**
Albumin (g/l)	122	38.6 ± 4.2	126	43.5 ± 3.0	**<0**.**001**
Bilirubin (µmol/l)	122	12 (9–17)	123	7 (5–10)	**<0**.**001**
ALP (IU/l)	122	418 (322–613)	126	564 (453–673)	**<0**.**001**
ALT (IU/l)	118	30 (22–61)	127	20 (15–29)	**<0**.**001**
AST (IU/l)	122	45 (34–72)	125	34 (27–39)	**<0**.**001**
GGT (IU/l)	118	23 (18–58)	124	15 (12–26)	**<0**.**001**
IgG (g/l)	110	13.2 ± 4.0	123	10.6 ± 4.1	**<0**.**001**
IgA (g/l)	110	2.1 (1.7–3.0)	123	1.7 (1.2–2.1)	**<0**.**001**
IgM (g/l)	110	1.3 (0.9–1.8)	123	1.2 (0.8–1.5)	**0**.**012**
**Ten Year Follow-up**	***N = 77*** *		***N = 107*** *		
Autoantibodies (% Positive)	69	45 (65%)	99	5 (5%)	**<0**.**001**
Albumin (g/l)	76	39.7 ± 4.0	104	43.2 ± 3.2	**<0**.**001**
Bilirubin (µmol/l)	77	10 (7–15)	104	8 (6–12)	0.073
ALP (IU/l)	76	433 (323–565)	104	408 (234–750)	0.740
ALT (IU/l)	72	26 (19–41)	105	19 (16–27)	**0**.**002**
AST (IU/l)	76	38 (32–47)	104	27 (24–35)	**<0**.**001**
GGT (IU/l)	72	24 (18–53)	104	16 (12–29)	**<0**.**001**
IgG (g/l)	69	13.3 ± 3.3	100	11.0 ± 3.4	**<0**.**001**
IgA (g/l)	69	2.2 (1.5–2.8)	100	2.0 (1.4–2.7)	0.309
IgM (g/l)	69	1.2 (0.8–1.6)	100	1.1 (0.9-1.5)	0.940

Data are reported as N (Column %), with *p*-values from Fisher's exact tests; median (interquartile range), with *p*-values from Mann-Whitney *U* tests; or as mean ± SD, with *p*-values from independent samples *t*-tests, as applicable. Bold *p*-values are significant at *p* < 0.05.

* The total number of patients assessed at the five/ten year follow up in each era. ALP, alkaline phosphatase; ALT, alanine aminotransferase; AST, aspartate aminotransferase; CSA, cyclosporine A; GGT, gamma-glutamyl transferase; IgG, immunoglobulin G; IgA, immunoglobulin A; IgM, immunoglobulin M; Pred, prednisolone; Tac, tacrolimus. The Tac + Pred cohort was significantly less likely to be autoantibody positive and displayed significantly lower levels of routine biochemical and immunological markers than the CSA-Only cohort at both time points.

### Auxological outcome

**Figure 2:**
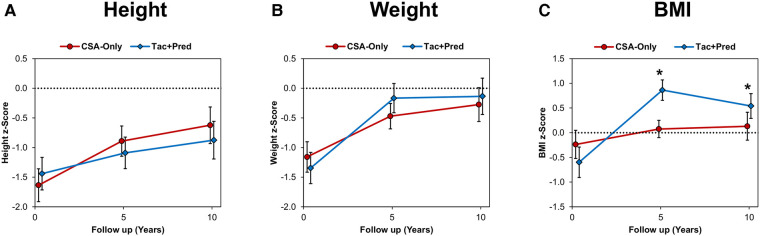
**Auxological outcomes compared between eras, (A) height, (B) weight, (C) BMI.** Points represent the mean z-scores at discharge and the five and ten year follow up assessments, with whiskers representing 95% confidence intervals. Broken lines are plotted at a z-score of zero. Comparisons between eras were performed using independent samples t-tests, which were non-significant (*p* < 0.05), unless indicated otherwise. There were no significant differences between eras in height or weight z-scores at either five or ten years. *BMI z-scores were significantly higher in patients from the Tac + Pred vs. CSA-Only era, with means of 0.86 vs. 0.07 (*p* < 0.001) and 0.54 vs. 0.13 (*p* = 0.039) at five and ten years, respectively. BMI, body mass index; CSA, cyclosporine A; Tac, tacrolimus; Pred, prednisolone.

Across the two eras, patients displayed impaired growth at discharge, with mean z-scores being significantly below zero for height (mean: −1.53 ± 1.56, *p* < 0.001), weight (-1.25 ± 1.49, *p* < 0.001) and BMI (-0.43 ± 1.69, *p* < 0.001). Comparisons between eras found no significant differences at the time of discharge in z-scores of height (*p* = 0.329), weight (*p* = 0.320), or BMI (*p* = 0.095). For both eras, the z-scores for all three auxological measures were found to increase significantly between discharge and the ten year follow up (all *p* < 0.05). There were no significant differences between eras in the z-scores for height or weight at either the five or ten year follow up (Supplementary Table B). However, the z-score for BMI was found to be significantly higher in patients in the Tac + Pred era at both the five (mean: 0.86 ± 1.18 vs. 0.07 ± 0.93, *p* < 0.001) and ten (0.54 ± 1.25 vs. 0.13 ± 1.12, *p* = 0.039) year follow up assessments (Figure 2; Supplementary Table B).

### Protocol biopsy findings

**Figure 3:**
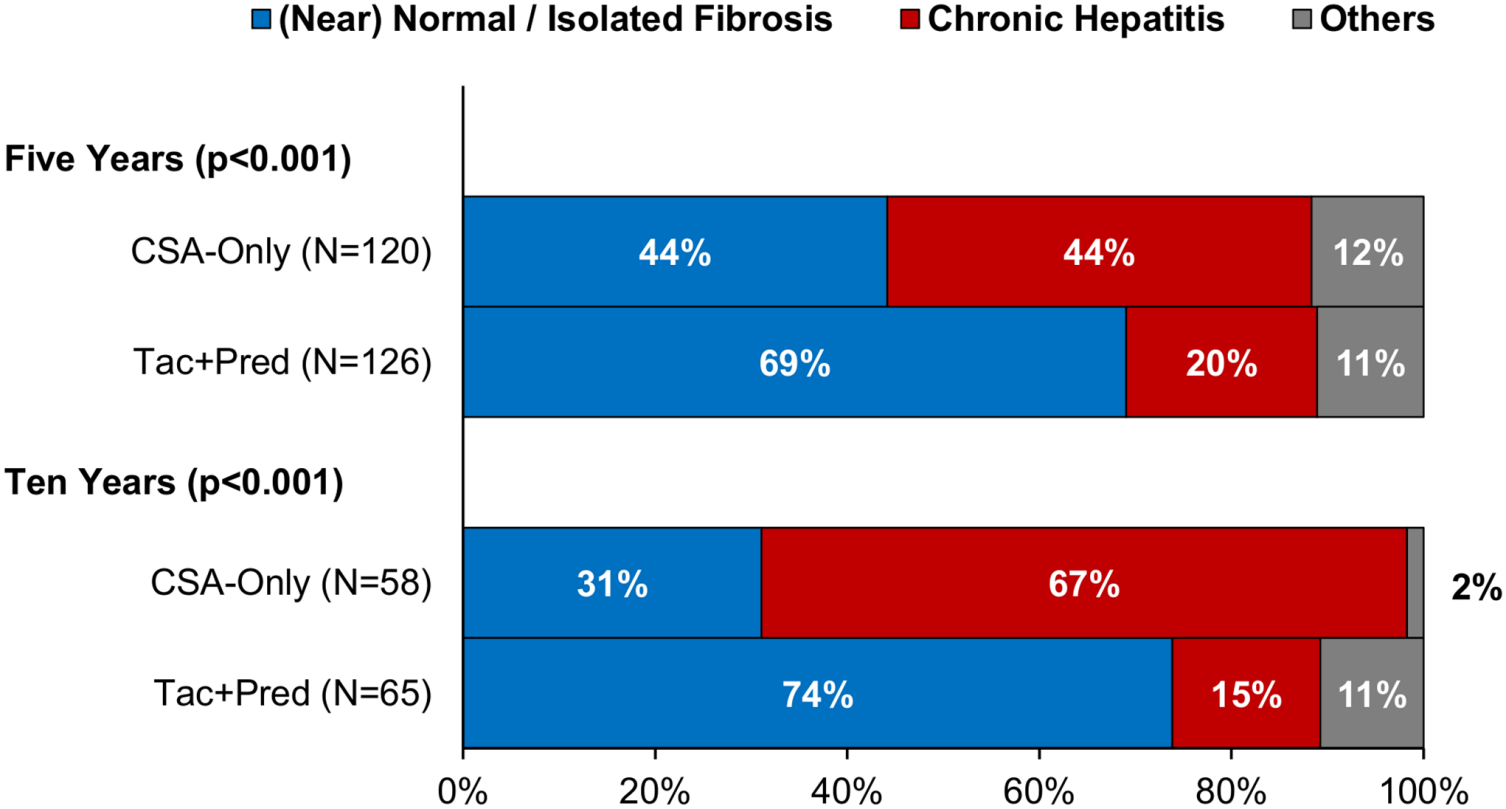
**Histological findings at five- and ten-year protocol biopsies by era.** Comparisons of the distribution of histological findings found significant differences between the eras at both five and ten years (Fisher's exact test: *p* < 0.001). In both cases, rates of chronic hepatitis were significantly reduced in the Tac + Pred era. CSA, cyclosporine A; Pred, prednisolone; Tac, tacrolimus.

**Figure 4:**
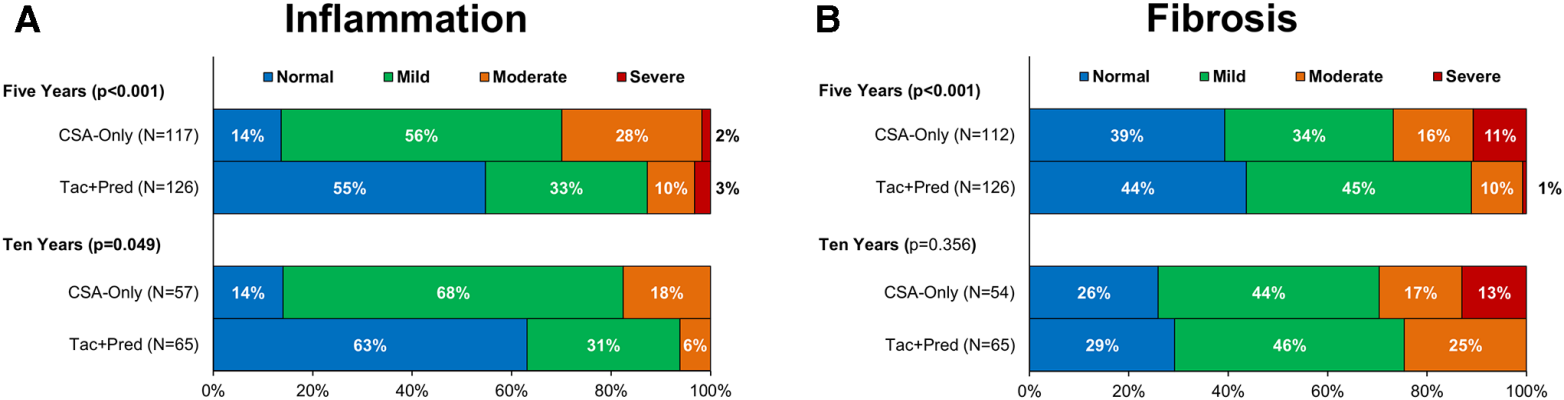
**Allograft inflammation (A) and fibrosis (B) at five- and ten-year protocol biopsies by era.** p-Values are from Mann-Whitney U tests, comparing the severity of inflammation/fibrosis between the two eras, and bold p-values are significant at *p* < 0.05. These found the Tac + Pred cohort to have significantly less severe inflammation than the CSA-Only cohort at both time points. Levels of fibrosis were found to be significantly lower in the Tac + Pred at the five-year biopsy, but not at ten years. CSA, cyclosporine A; Pred, prednisolone; Tac, tacrolimus.

At the five-year protocol biopsy, histological findings differed significantly between eras (*p* < 0.001, Figure 3). In the Tac + Pred era, 69% of biopsies were near normal or had isolated fibrosis, compared to 44% of those in the earlier CSA-Only era, with 20% vs. 44% having signs of CH. This difference between eras persisted at the ten-year biopsy (*p* < 0.001). Similar trends were observed for inflammation, with moderate-severe inflammation observed in 13% vs. 30% (*p* < 0.001) of patients in the Tac + Pred vs. CSA-Only eras at the five-year protocol biopsy, and 6% vs. 18% (*p* = 0.049) at the ten-year protocol biopsy (Figure 4A). Fibrosis was also found to be significantly less advanced in patients from the Tac + Pred era at the five-year protocol biopsy (moderate-severe: 11% vs. 27%, *p* < 0.001), although this difference was not significant at the ten-year biopsy (25% vs. 30%, *p* = 0.356) (Figure 4B).

These comparisons were limited by the fact that several baseline variables were found to differ significantly between the Tac + Pred vs. CSA-Only eras, as previously described (Table 2), which may have acted as confounding factors. As such, multivariable analyses were performed for the five-year protocol biopsy outcomes, with inflammation and fibrosis dichotomized as moderate/severe vs. none/mild, and histological findings as CH vs. near normal/isolated fibrosis. Further details about the methodology used, and the resulting models are reported in Supplementary Table C. These models found no significant associations between five-year biopsy outcomes and either donor/recipient age, graft type or CIT, implying that the differences in histological findings between the Tac + Pred vs. CSA-Only eras were likely independent of these factors.

### Association between graft inflammation and fibrosis

**Figure 5:**
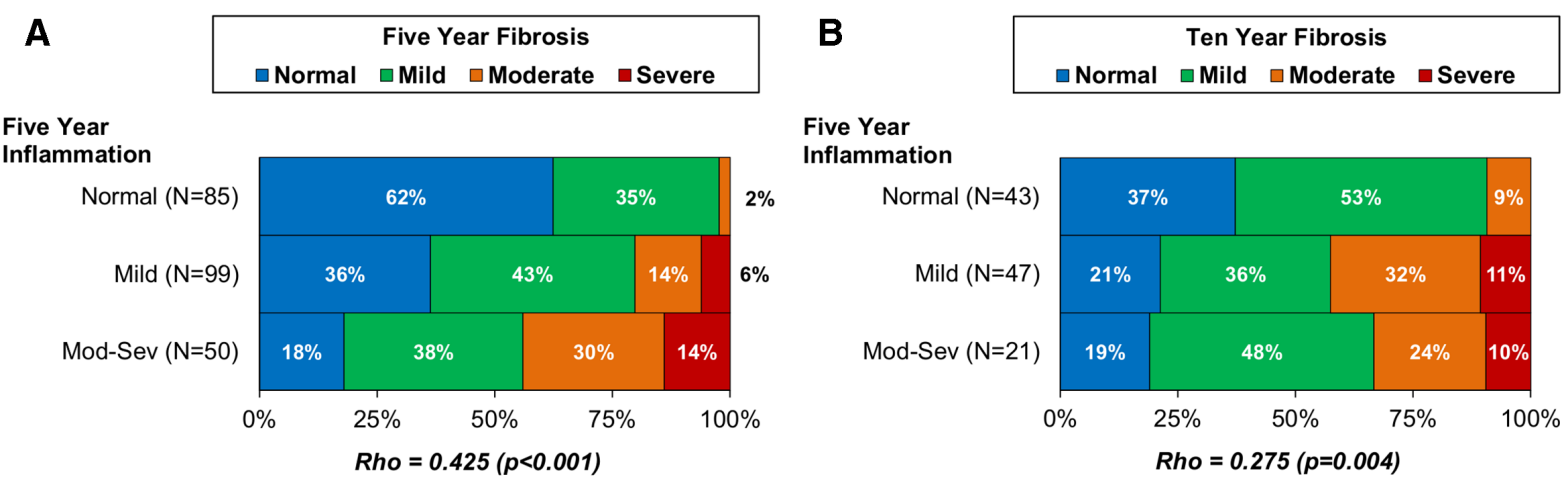
**Association of five-year allograft inflammation with fibrosis at five-year (A) and ten-year (B) protocol biopsy in the combined CSA-Only and Tac + Pred cohorts.** *Associations between the degrees of inflammation and fibrosis were assessed using Spearman's (rho) correlation coefficients, and bold p-values are significant at p < 0.05. These found the degree of inflammation at five years to be significantly associated with the degree of fibrosis at five-year biopsy, and with the presence of fibrosis at 10 years. Pred, prednisolone; Tac, tacrolimus*.

In order to further investigate the role of inflammation as a driver of fibrogenesis, the association of five-year graft inflammation with fibrosis at five and ten years was assessed for the combined cohort. The level of fibrosis at five years was found to increase progressively with the corresponding level of inflammation (Spearman's rho: 0.425, *p* < 0.001, Figure 5A). A significant correlation between five-year inflammation and ten year fibrosis was also observed (Spearman's rho: 0.275, *p* = 0.004, Figure 5B). However, further assessment found the degree of fibrosis at ten years to be similar in those with mild and moderate-severe inflammation at five years. As such, this correlation was largely driven by a threshold effect, where having any inflammation at five years was associated with a greater degree of fibrosis at ten years, rather than a progressive increase in fibrosis with increasing inflammation.

### Further predictors of fibrosis and inflammation

For the Tac + Pred cohort, associations between cohort characteristics and the degree of inflammation and fibrosis at five and ten years were then assessed and are available as supplementary tables (Supplementary Table D and E for associations with inflammation at five and ten years, respectively; Supplementary Table F and G for associations with fibrosis at five and ten years, respectively). These did not identify any meaningful associations, with the only significant finding being a significantly higher baseline recipient height in those with greater five-year allograft inflammation (*p* = 0.021). None of the other demographic- or transplant-related factors were found to be significantly associated with any of the outcomes considered.

## Discussion

We have completed a unique retrospective, single-centre cohort study comparing the outcome of protocol assessments, including protocol liver biopsies, between two eras of IS: one cohort using a combination of tacrolimus and low-dose maintenance prednisolone (Tac + Pred) and a historical cohort using a steroid-free, cyclosporine-based protocol (CSA-Only).

We have previously reported chronic hepatitis (CH) in a cohort of patients with a steroid-free maintenance IS regimen (6). CH increased in prevalence with time since transplantation, affecting nearly half of children after five years and close to 2/3 of children by ten years, and was accompanied by worsening fibrosis. Traditional surrogate markers of hepatocellular damage, such as transaminases, were not helpful in identifying these histological changes (6). Other transplant centres have reported similar findings, and found these changes were progressive (3, 13, 16–18). The exact pathophysiological mechanism for CH has remained unclear (19). Our hypothesis is that CH is part of a spectrum of alloimmune inflammation and rejection, and can respond to immunosuppressive treatment.

We have shown that increased immunosuppression is associated with a decrease in the incidence of CH at five- and ten-years post-transplantation. This is mirrored by a lower degree of histological inflammatory activity at both time points and is accompanied by a significant decrease in autoantibody positivity, immunoglobulin levels, median transaminases, compared to the CSA-Only cohort.

Other studies complement our evidence regarding the impact of immunosuppression on CH following paediatric transplantation. Kosola et al. showed that the use of low-dose corticosteroids was associated with fewer and milder histological changes during long-term follow-up. However, in this study the biopsies were not scheduled at standardized time points, which complicates interpretation of histology (20). Pongpaibul et al. described a group of children with established *de novo* autoimmune hepatitis, and showed reduction in inflammatory activity and improved liver function following treatment with corticosteroids and Mycophenolate (21).

Our findings are also supported by a study which correlated histopathological findings in late post-transplant biopsies with gene expression profiling findings in liver tissue and blood, and found CH to have a similar gene expression signature to T-cell mediated rejection (22, 23). Feng et al. have subsequently supported this by identifying an association of a transcriptome overexpressed in T-cell mediated rejection with a cluster of patients with portal and interface inflammation, but not with patients with fibrosis-only or normal/near normal five year biopsies (3). Vionnet et al. further described a cohort of post-transplant patients who displayed clinically-silent inflammatory allograft changes which were associated with overexpression of a TCMR-related genetic profile and reduced Tacrolimus-exposure (24).

Inflammation appears to have been an important driver of fibrosis in our study, with significant correlation between the degree of five-year graft inflammation, and both the degree of fibrosis at five years and the presence of fibrosis at ten years post-transplant (*p* < 0.001 and *p* = 0.004 respectively). Increased immunosuppression in the Tac + Pred era was also associated with a reduced prevalence of fibrosis at five years. This finding is supported in a recent multicentre study by Junge at al, which demonstrated an association of Prednisolone-free IS with allograft fibrosis (25). Ruth et al. underline the role of alloimmunity in the evolution of silent allograft fibrosis (26). Importantly, this effect was only significant five years post-transplant in our study, suggesting that long-term hepatic allograft fibrogenesis is a multifactorial process (27) including both immunological (28–30) and non-immunological factors (16, 31–35). Contrary to other groups, we were not able establish an association of graft fibrosis with demographic or transplant-related factors, and the rates of biliary and vascular complications did not differ significantly between both eras.

The differences between studies with view to fibrosis-related variables and factors indicate that the degree of graft fibrosis is not merely the result of the stepwise, unidirectional deposition of extracellular matrix components rather than the result of a dynamic process of fibrogenic and fibro-degradative mechanisms (36). The complexity of interacting factors involved is slowly unravelling (37). Corticosteroids, for example, have been demonstrated to have a dual effect on liver fibrosis in animal experiments, on the one hand dampening fibrogenic gene expression in hepatic stellate cells (HSC), whilst on the other hand exacerbating liver fibrosis (38). The indiscriminate suppression of a recently newly identified subpopulation of CD8-positive lymphocytes who promote HSC-cell apoptosis may potentially play a role (39).

The underlying immunological mechanisms of CH are still unclear. The fact that there is no measurable difference in acute TCMR or chronic rejection between both eras in our study might reflect the small prednisolone dose used in the Tac-Pred cohort not reaching the necessary dose threshold to prevent and contain typical rejection. It also suggests that the pathway of allorecognition of “classical” TCMR is different from the processes involved in the more indolent changes observed in CH (24). Also, a role of the humoral immune system and antibody-mediated rejection has been postulated (3, 14, 24). Feng et al. linked class II DSAs with the expression of a number of cytokines preceding hepatic T-cell infiltration (3). Burns et al. described the ability of alloantibodies to activate alloreactive *T*-cells in patients following skin and heart transplantation (40). Vionnet et al. suggested that the fibro-inflammatory graft changes may involve an alloreactive process enhanced by DSAs (24). It is likely that “chronic hepatitis” represents a form of late rejection, either manifesting as late TCMR and/or chronic ABMR.

Growth impairment and reduced bone mineral density are recognized post-transplant complications in paediatric liver transplantation, and their aetiology is multifactorial (41). Long-term corticosteroid exposure predisposes to developing metabolic syndrome, growth impairment and weight gain (42). Importantly, in our cohort, long-term, low-dose exposure to prednisolone did not significantly affect height z-scores at five and ten years post–transplantation, compared to the CSA-Only cohort. However, significantly higher z-scores for BMI were observed in the Tac + Pred group at five and ten year follow ups, suggesting that the long-term prednisolone exposure may have resulted in some excess weight gain.

It is of course important to note that the results of this study will not justify the indiscriminate increase of post-transplant immunosuppression. Nephrotoxicity, cardiovascular side effects or other extrahepatic immunosuppression-related morbidity must be balanced against the benefit of reduced allograft inflammation (24, 43). Longitudinal prospective clinical and allograft outcome data in combination with “next generation pathology” techniques and gene and protein expression profiles in blood and tissue will help to individualise future immunosuppression protocols and facilitate tolerance prediction models (44, 45).

The main strength of this study is the large, well-defined cohort of patients with meticulous long-term follow-up. The limitations are mostly related to the retrospective design of the study, which makes it difficult to rigorously control both treatment eras. Primarily, the two treatment eras did not only differ in the use of Prednisolone, but also in the type of first-line (i.e., type of calcineurin inhibitor) and the rate of second-line immunosuppression (Azathioprine, MMF). There are also other era-related differences, such as the more frequent use of split grafts from older donors in younger recipients in the Tac + Pred era, compared to CSA-Only. Due to the magnitude of these differences, it was not possible to perform a case-matched analysis to negate the differences in cohort characteristics. Whilst a multivariable analysis was attempted for the five-year protocol biopsy outcomes, it was not possible to produce a comprehensive model, given the sample size and small number of outcomes, meaning that residual confounding may have persisted. As such, it is possible that these other changes in cohort characteristics may have influenced the main comparison between treatment eras. Secondly, other more detailed scores for assessment of liver allograft fibrosis are now available such as the one by Venturi et al. (31) We have used the same semi-quantitative histological assessment score for quantifying inflammation and fibrosis as Evans et al. (6) which allowed us to maintain consistency in terminology used in the earlier era of the study. Efforts to standardise histopathological assessment for future prospective, longitudinal studies are ongoing (46). Finally, the exclusion criteria used will potentially have introduced some degree of selection bias, particularly with respect to two specific excluded subgroups. The first of these was those patients who either died or were re-transplanted prior to five years, who were excluded since a protocol biopsy was not possible. These patients likely represented a subset with more severe post-transplant complications; hence, the disproportionate proportion of these exclusions from the CSA-Only cohort may have resulted in an underestimate of the degree of histological changes in this era. However, since histological outcomes were found to be significantly better in the Tac + Pred era, it is likely that any such selection bias will have resulted in an underestimate of the underlying difference between eras; consequently, we feel that the conclusions of the study would be unaffected.

The second subgroup of patients were those from the CSA-Only era who restarted prednisolone between the five- and ten-year follow-up assessments; these patients were excluded since they deviated from the standard IS of the era. However, since 10-year biopsies were available for the majority of these excluded patients, an intention-to-treat analysis was also performed, which additionally included the patients from the CSA-Only cohort who had restarted prednisolone. This returned consistent results to the primary analysis for the histological outcomes (Supplementary Table H) and did not change the conclusions of the study.

In conclusion, we have demonstrated that the development of chronic graft hepatitis may be effectively reduced by increased IS, whilst this only partially impacts long-term allograft fibrosis. This suggests that CH is an immune-mediated process, which may be a manifestation of atypical rejection, whilst fibrosis is of multifactorial origin.

## Data Availability

The raw data supporting the conclusions of this article will be made available by the authors, without undue reservation.
