# Supplementary material for: The role of immunosuppression in long-term graft hepatitis and fibrosis after paediatric liver transplant – comparison of two treatment protocols
Source: Front Transplant. 2023 Feb 28;1:1042676. doi: 10.3389/frtra.2022.1042676 (PMC11235287; doi:10.3389/frtra.2022.1042676)
Supplement: Supplementary file 1 [file Table1.docx]

# SUPPORTING INFORMATION (DESCRIPTION)

# TABLE OF CONTENT

Supporting information includes eight supplementary tables:

- Supplementary Table A summarises the baseline cohort characteristics by era for the subgroup of patients with a ten-year protocol biopsy.
- Supplementary Table B compares the auxological outcome in both eras at transplant episode discharge, five and ten years post transplantation.
- Supplementary Table C presents the multivariable analyses of outcomes on five-year protocol biopsy.
- Supplementary Table D and E summarise the analyses of factors associated with inflammation at five- and ten-year protocol biopsy in the Tac+Pred cohort.
- Supplementary Tables F and G summarise the analyses of factors associated with fibrosis at five- and ten-year protocol biopsy in the Tac+Pred cohort.
- Supplementary Table H summarises the results of an intention-to-treat analysis for the ten-year protocol biopsy including N=12 patients who had been restarted on Prednisolone following the five-year protocol biopsy.

# TITLE

The role of immunosuppression in long-term graft hepatitis and fibrosis after paediatric liver transplant – comparison of two treatment protocols

# AUTHORS

Wolfram Haller

ORCID 0000-0002-0518-7383

Department of Gastroenterology&Nutrition

Birmingham Woman’s and Children’s Hospital NHS Foundation Trust

Birmingham, United Kingdom

Institute of Clinical Sciences

University of Birmingham

Birmingham, United Kingdom

Email [wolframhaller@nhs.net](mailto:wolframhaller@nhs.net) ; [W.haller@bham.ac.uk](mailto:W.haller@bham.ac.uk)

James Hodson

ORCID 0000-0003-3487-0090

Institute of Translational Medicine

University Hospitals Birmingham NHS Foundation Trust

Birmingham, United Kingdom

Email [James.Hodson@uhb.nhs.uk](mailto:James.Hodson@uhb.nhs.uk)

Rachel Brown

Department of Cellular Pathology

Queen Elizabeth Hospital

Birmingham, United Kingdom

Email [Rachel.Brown@uhb.nhs.uk](mailto:Rachel.Brown@uhb.nhs.uk)

Carla Lloyd

The Liver Unit

Birmingham Woman’s and Children’s Hospital NHS Foundation Trust

Birmingham, United Kingdom

Email [carla.lloyd1@nhs.net](mailto:carla.lloyd1@nhs.net)

Stefan Hubscher

ORCID 0000-0003-1609-8527

Department of Cellular Pathology

Queen Elizabeth Hospital

Birmingham, United Kingdom

Institute for Immunology and Immunotherapy

University of Birmingham

Birmingham, United Kingdom

Email [S.G.HUBSCHER@bham.ac.uk](mailto:S.G.HUBSCHER@bham.ac.uk)

Patrick McKiernan

ORCID 0000-0002-8466-8504

The Liver Unit

Birmingham Woman’s and Children’s Hospital NHS Foundation Trust

Birmingham, United Kingdom

Email [patmckiernan@hotmail.com](mailto:patmckiernan@hotmail.com)

Deidre Kelly

ORCID 0000-0001-9776-4354

The Liver Unit

Birmingham Women's and Children's NHS Foundation Trust

Birmingham, UK

Institute for Immunology and Immunotherapy

University of Birmingham

Birmingham, United Kingdom

Email [deirdre@kellyda.co.uk](mailto:deirdre@kellyda.co.uk); [deirdrekelly@nhs.net](mailto:deirdrekelly@nhs.net)

**Supplementary Table A - Baseline cohort characteristics by era for the subgroup of patients with a ten-year protocol biopsy**

|  | **CSA-Only** | | **Tac+Pred** | | **p-Value** |
| --- | --- | --- | --- | --- | --- |
|  | **N** | ***Statistic*** | **N** | ***Statistic*** |  |
| Age (Years) |  |  |  |  |  |
| *Recipient* | 58 | 1.6 (0.8-4.6) | 67 | 1.4 (0.8-3.1) | 0.362 |
| *Donor* | 58 | 9.2 (6.0-14.0) | 66 | 18.0 (11.3-29.7) | **<0.001** |
| Gender |  |  |  |  |  |
| *Recipient (% Male)* | 58 | 24 (41%) | 67 | 39 (58%) | 0.074 |
| *Donor (% Male)* | 58 | 36 (62%) | 64 | 42 (66%) | 0.709 |
| *Donor/Recipient Match* | 58 | 30 (52%) | 64 | 32 (50%) | 0.858 |
| Diagnosis | 58 |  | 67 |  | 0.614** |
| *Biliary Atresia* |  | 33 (57%) |  | 39 (58%) |  |
| *Metabolic* |  | 10 (17%) |  | 10 (15%) |  |
| *Acute Liver Failure* |  | 5 (9%) |  | 5 (7%) |  |
| *Cholestasis* |  | 5 (9%) |  | 3 (4%) |  |
| *Malignancy* |  | 0 (0%) |  | 2 (3%) |  |
| *Autoimmune Liver Disease* |  | 0 (0%) |  | 1 (1%) |  |
| *Other* |  | 0 (0%) |  | 2 (3%) |  |
| *Indeterminate* |  | 5 (9%) |  | 5 (7%) |  |
| CMV Serostatus |  |  |  |  |  |
| *Recipient (% Positive)* | 57 | 16 (28%) | 67 | 18 (27%) | 1.000 |
| *Donor (% Positive)* | 56 | 24 (43%) | 67 | 21 (31%) | 0.195 |
| Blood Group Mismatch | 57 |  | 64 |  | 0.557* |
| *No* |  | 51 (89%) |  | 55 (86%) |  |
| *Minor* |  | 6 (11%) |  | 9 (14%) |  |
| *Major (ABOi)* |  | 0 (0%) |  | 0 (0%) |  |
| Graft Type | 58 |  | 67 |  | **<0.001** |
| *Whole* |  | 21 (36%) |  | 13 (19%) |  |
| *Split* |  | 0 (0%) |  | 29 (43%) |  |
| *Reduced* |  | 37 (64%) |  | 25 (37%) |  |
| CIT (Minutes) | 58 | 638 ± 214 | 61 | 602 ± 122 | 0.269 |

*Analyses include only those patients who underwent a ten-year protocol biopsy (N=125). Data are reported as N (Column %), with p-values from Fisher's exact tests; median (interquartile range), with p-values from Mann-Whitney U tests; or as mean ± SD, with p-values from independent samples t-tests, unless stated otherwise. Bold p-values are significant at p<0.05. *p-Value from Mann-Whitney U test, as the factor is ordinal. **p-Value from Chi-square test, as Fisher's exact test was incalculable. ABOi, ABO incompatible; BMI, body mass index; CMV, cytomegalovirus; CIT, cold ischaemic time; CSA, cyclosporine A; Pred, prednisolone; Tac, tacrolimus.*

**Supplementary Table B – Auxological outcomes by era**

|  | **CSA-Only** | | **Tac+Pred** | | **p-Value**  **(Eras)**** |
| --- | --- | --- | --- | --- | --- |
|  | ***N*** | ***Statistic*** | ***N*** | ***Statistic*** |  |
| Height z-Score |  |  |  |  |  |
| *Discharge* | 116 | -1.64 ± 1.52 | 128 | -1.44 ± 1.58 | 0.329 |
| *Five Years* | 109 | -0.89 ± 1.36 | 124 | -1.09 ± 1.51 | 0.294 |
| *Ten Years* | 61 | -0.62 ± 1.23 | 96 | -0.88 ± 1.59 | 0.294 |
| *p-Value (0 vs. 10 Years)** | ***p<0.001*** | | ***p<0.001*** | |  |
| Weight z-Score |  |  |  |  |  |
| *Discharge* | 125 | -1.16 ± 1.46 | 128 | -1.35 ± 1.52 | 0.320 |
| *Five Years* | 110 | -0.47 ± 1.15 | 126 | -0.17 ± 1.41 | 0.073 |
| *Ten Years* | 61 | -0.27 ± 1.14 | 98 | -0.14 ± 1.55 | 0.544 |
| *p-Value (0 vs. 10 Years)** | ***p<0.001*** | | ***p<0.001*** | |  |
| BMI z-Score |  |  |  |  |  |
| *Discharge* | 115 | -0.24 ± 1.56 | 128 | -0.60 ± 1.78 | 0.095 |
| *Five Years* | 109 | 0.07 ± 0.93 | 125 | 0.86 ± 1.18 | **<0.001** |
| *Ten Years* | 61 | 0.13 ± 1.12 | 96 | 0.54 ± 1.25 | **0.039** |
| *p-Value (0 vs. 10 Years)** | ***p=0.042*** | | ***p<0.001*** | |  |

*Data are reported as mean ± standard deviation, and bold p-values are significant at p<0.05. *p-Value from paired t-tests, comparing between discharge and ten years. **p-Values from independent samples t-tests, comparing between eras.* *BMI, body mass index; CSA, cyclosporine A; Pred, prednisolone; Tac, tacrolimus.*

**Supplementary Table C – Multivariable analyses of outcomes on five-year protocol biopsy**

|  | ***Moderate/Severe Inflammation*** | | ***Moderate/Severe Fibrosis*** | | ***Chronic Hepatitis*** | |
| --- | --- | --- | --- | --- | --- | --- |
| **Factor** | *Odds Ratio (95% CI)* | *p-Value* | *Odds Ratio (95% CI)* | *p-Value* | *Odds Ratio (95% CI)* | *p-Value* |
| **Univariable Models** | | | | | | |
| Cohort (Tac+Pred) | 0.34 (0.18-0.66) | **0.001** | 0.34 (0.17-0.69) | **0.002** | 0.29 (0.16-0.52) | **<0.001** |
| **Multivariable Models** | | | | | | |
| Cohort (Tac+Pred) | 0.44 (0.18-1.06) | 0.066 | 0.34 (0.12-0.96) | **0.042** | 0.44 (0.20-0.96) | **0.038** |
| Recipient Age (per Year) | 0.94 (0.86-1.04) | 0.234 | 1.00 (0.90-1.10) | 0.974 | 1.06 (0.97-1.15) | 0.203 |
| Donor Age (per Year) | 1.01 (0.98-1.04) | 0.602 | 0.99 (0.96-1.02) | 0.639 | 1.00 (0.97-1.02) | 0.765 |
| Graft Type |  | 0.057 |  | 0.774 |  | 0.503 |
| *Whole* | *Reference* | - | *Reference* | - | *Reference* | - |
| *Split* | 0.22 (0.06-0.84) | **0.026** | 1.34 (0.34-5.21) | 0.674 | 0.62 (0.20-1.89) | 0.401 |
| *Reduced* | 0.41 (0.17-1.04) | 0.061 | 0.88 (0.32-2.43) | 0.807 | 1.08 (0.46-2.52) | 0.861 |
| Cold Ischaemic Time (per Hour) | 0.99 (0.89-1.10) | 0.823 | 1.08 (0.96-1.21) | 0.204 | 1.02 (0.92-1.13) | 0.711 |

*Results are from binary logistic regression models, with five-year protocol biopsy outcomes dichotomised for analysis. Inflammation and fibrosis were grouped as moderate/severe vs. none/mild, whilst the histological findings were grouped as chronic hepatitis vs. (near) normal/isolated fibrosis, with patients in the “others” category excluded from analysis. Initially, univariable analyses were produced for each outcome, with the cohort (Tac+Pred vs. CSA-Only) as the only covariate. Multivariable models were then produced, which additionally included the factors found to differ significantly between the Tac+Pred vs. CSA-Only cohorts in Table 2 as covariates. After excluding patients with missing data for any of the factors considered, these multivariable models were based on N=230 (N=48 outcomes) for inflammation, N=225 (N=43 outcomes) for fibrosis, and N=206 (N=76 outcomes) for chronic hepatitis. Age and cold ischaemic time were treated as continuous variables, with odds ratios reported per unit increase, whilst the cohort and graft type were treated as nominal, with odds ratios reported for the stated category, relative to the reference category. Bold p-values are significant at p<0.05. Pred, prednisolone; Tac, tacrolimus.*

**Supplementary Table D - Associations with inflammation at five years in the Tac+Pred cohort.**

|  | **Five Year Inflammation** | | | |
| --- | --- | --- | --- | --- |
|  | ***N*** | ***None/Mild*** | ***Moderate/Severe*** | ***p-Value*** |
| Recipient Age (Years) | 126 | 1.9 (0.8-5.1) | 2.4 (1.3-10.3) | 0.957 |
| Donor Age (Years) | 126 | 22.9 (14.8-35.2) | 18.0 (12.3-27.2) | 0.961 |
| Recipient Gender |  |  |  | 0.163 |
| *Male* | 54 | 45 (83%) | 9 (17%) |  |
| *Female* | 72 | 65 (90%) | 7 (10%) |  |
| Donor Gender |  |  |  | 0.670 |
| *Male* | 85 | 74 (87%) | 11 (13%) |  |
| *Female* | 39 | 34 (87%) | 5 (13%) |  |
| Donor/Recipient Gender Match |  |  |  | 0.339 |
| *No* | 66 | 60 (91%) | 6 (9%) |  |
| *Yes* | 58 | 48 (83%) | 10 (17%) |  |
| Recipient Height (z-Score) | 126 | -1.52 ± 1.61 | -0.95 ± 1.39 | **0.021** |
| Recipient Weight (z-Score) | 126 | -1.42 ± 1.48 | -0.89 ± 1.80 | 0.073 |
| Recipient BMI (z-Score) | 126 | -0.60 ± 1.78 | -0.60 ± 1.95 | 0.550 |
| Diagnosis |  |  |  | 0.933 |
| *BA/Cholestasis* | 64 | 57 (89%) | 7 (11%) |  |
| *Metabolic* | 22 | 18 (82%) | 4 (18%) |  |
| *ALF* | 17 | 14 (82%) | 3 (18%) |  |
| *Others* | 23 | 21 (91%) | 2 (9%) |  |
| Recipient CMV Serostatus |  |  |  | 0.800 |
| *Negative* | 91 | 83 (91%) | 8 (9%) |  |
| *Positive* | 33 | 25 (76%) | 8 (24%) |  |
| Donor CMV Serostatus |  |  |  | 0.639 |
| *Negative* | 76 | 67 (88%) | 9 (12%) |  |
| *Positive* | 47 | 40 (85%) | 7 (15%) |  |
| Blood Group Mismatch |  |  |  | 0.524* |
| *No* | 106 | 93 (88%) | 13 (12%) |  |
| *Minor* | 15 | 12 (80%) | 3 (20%) |  |
| *Major* | 3 | 3 (100%) | 0 (0%) |  |
| Graft Type |  |  |  | 0.284 |
| *Whole* | 18 | 12 (67%) | 6 (33%) |  |
| *Split* | 68 | 64 (94%) | 4 (6%) |  |
| *Reduced* | 40 | 34 (85%) | 6 (15%) |  |
| CIT (Minutes) | 113 | 595 ± 122 | 541 ± 98 | 0.050 |
| Biliary Complications | 126 |  |  | 0.162 |
| *No* |  | 99 (88%) | 14 (12%) |  |
| *Yes* |  | 11 (85%) | 2 (15%) |  |
| Vascular Complication | 126 |  |  | 0.696 |
| *No* |  | 99 (88%) | 14 (12%) |  |
| *Yes* |  | 11 (85%) | 2 (15%) |  |
| Any Acute TCMR | 126 |  |  | 0.889 |
| *No* |  | 51 (89%) | 6 (11%) |  |
| *Yes* |  | 59 (86%) | 10 (14%) |  |
| Autoantibodies (Five Years) | 122 |  |  | 0.773 |
| *Negative* |  | 90 (89%) | 11 (11%) |  |
| *Positive* |  | 17 (81%) | 4 (19%) |  |
| Autoantibodies (Ten Years) | 97 |  |  | 0.649 |
| *Negative* |  | 83 (90%) | 9 (10%) |  |
| *Positive* |  | 5 (100%) | 0 (0%) |  |

*Continuous factors are reported as median (interquartile range) or mean ± SD, with p-values from Spearman’s correlation coefficients. Nominal factors are reported as N (Row %), with p-values from Mann-Whitney U or Kruskal-Wallis tests, unless stated otherwise. All p-values were generated using all four categories of the inflammation/fibrosis scores, which were subsequently combined to none/mild and moderate/severe for reporting in the table for brevity. Bold p-values are significant at p<0.05. *p-Value from Spearman’s correlation coefficient, as the factor is ordinal. acute TCMR, acute T-cell mediated rejection; ALF, acute liver failure; BA, biliary atresia; BMI, body mass index; CMV, cytomegalovirus; CIT, cold ischemic time; Pred, prednisolone; Tac, tacrolimus.*

**Supplementary Table E - Associations with inflammation at ten years in the Tac+Pred cohort.**

|  | **Ten Year Inflammation** | | | |
| --- | --- | --- | --- | --- |
|  | ***N*** | ***None/Mild*** | ***Moderate/Severe*** | ***p-Value*** |
| Recipient Age (Years) | 65 | 1.5 (0.8-3.1) | 0.8 (0.6-3.1) | 0.332 |
| Donor Age (Years) | 64 | 18.0 (12.2-28.7) | 42.8 (22.2-49.7) | 0.526 |
| Recipient Gender |  |  |  | 0.261 |
| *Male* | 39 | 37 (95%) | 2 (5%) |  |
| *Female* | 26 | 24 (92%) | 2 (8%) |  |
| Donor Gender |  |  |  | 0.899 |
| *Male* | 40 | 36 (90%) | 4 (10%) |  |
| *Female* | 22 | 22 (100%) | 0 (0%) |  |
| Donor/Recipient Gender Match |  |  |  | 0.810 |
| *No* | 30 | 28 (93%) | 2 (7%) |  |
| *Yes* | 32 | 30 (94%) | 2 (6%) |  |
| Recipient Height (z-Score) | 65 | -1.75 ± 1.49 | 0.02 ± 2.36 | 0.813 |
| Recipient Weight (z-Score) | 65 | -1.50 ± 1.46 | -0.40 ± 1.61 | 0.829 |
| Recipient BMI (z-Score) | 65 | -0.43 ± 1.84 | -0.41 ± 1.01 | 0.496 |
| Diagnosis |  |  |  | 0.863 |
| *BA/Cholestasis* | 40 | 37 (93%) | 3 (8%) |  |
| *Metabolic* | 10 | 10 (100%) | 0 (0%) |  |
| *ALF* | 5 | 4 (80%) | 1 (20%) |  |
| *Others* | 10 | 10 (100%) | 0 (0%) |  |
| Recipient CMV Serostatus |  |  |  | 0.080 |
| *Negative* | 47 | 44 (94%) | 3 (6%) |  |
| *Positive* | 18 | 17 (94%) | 1 (6%) |  |
| Donor CMV Serostatus |  |  |  | 1.000 |
| *Negative* | 45 | 43 (96%) | 2 (4%) |  |
| *Positive* | 20 | 18 (90%) | 2 (10%) |  |
| Blood Group Mismatch |  |  |  | 0.334* |
| *No* | 53 | 49 (92%) | 4 (8%) |  |
| *Minor* | 9 | 9 (100%) | 0 (0%) |  |
| *Major* | 0 | 0 (0%) | 0 (0%) |  |
| Graft Type |  |  |  | 0.286 |
| *Whole* | 12 | 11 (92%) | 1 (8%) |  |
| *Split* | 29 | 27 (93%) | 2 (7%) |  |
| *Reduced* | 24 | 23 (96%) | 1 (4%) |  |
| CIT (Minutes) | 59 | 601 ± 116 | 601 ± 182 | 0.070 |
| Biliary Complications | 65 |  |  | 0.937 |
| *No* |  | 57 (93%) | 4 (7%) |  |
| *Yes* |  | 4 (100%) | 0 (0%) |  |
| Vascular Complication | 65 |  |  | 0.623 |
| *No* |  | 53 (93%) | 4 (7%) |  |
| *Yes* |  | 8 (100%) | 0 (0%) |  |
| Any Acute TCMR | 65 |  |  | 0.877 |
| *No* |  | 27 (96%) | 1 (4%) |  |
| *Yes* |  | 34 (92%) | 3 (8%) |  |
| Autoantibodies (Five Years) | 64 |  |  | 0.738 |
| *Negative* |  | 43 (93%) | 3 (7%) |  |
| *Positive* |  | 17 (94%) | 1 (6%) |  |
| Autoantibodies (Ten Years) | 60 |  |  | 0.385 |
| *Negative* |  | 54 (95%) | 3 (5%) |  |
| *Positive* |  | 3 (100%) | 0 (0%) |  |

*Continuous factors are reported as median (interquartile range) or mean ± SD, with p-values from Spearman’s correlation coefficients. Nominal factors are reported as N (Row %), with p-values from Mann-Whitney U or Kruskal-Wallis tests, unless stated otherwise. All p-values were generated using all four categories of the inflammation/fibrosis scores, which were subsequently combined to none/mild and moderate/severe for reporting in the table for brevity. Bold p-values are significant at p<0.05. *p-Value from Spearman’s correlation coefficient, as the factor is ordinal. acute TCMR, acute T-cell mediated rejection; ALF, acute liver failure; BA, biliary atresia; BMI, body mass index; CMV, cytomegalovirus; CIT, cold ischemic time; Pred, prednisolone; Tac, tacrolimus.*

**Supplementary Table F - Associations with fibrosis at five years in the Tac+Pred cohort.**

|  | **Five Year Fibrosis** | | | |
| --- | --- | --- | --- | --- |
|  | ***N*** | ***None/Mild*** | ***Moderate/Severe*** | ***p-Value*** |
| Recipient Age (Years) | 126 | 2.0 (0.8-5.3) | 1.1 (0.7-4.2) | 0.258 |
| Donor Age (Years) | 126 | 22.1 (13.8-33.8) | 22.1 (15.2-36.8) | 0.124 |
| Recipient Gender |  |  |  | 0.220 |
| *Male* | 54 | 49 (91%) | 5 (9%) |  |
| *Female* | 72 | 63 (88%) | 9 (13%) |  |
| Donor Gender |  |  |  | 0.217 |
| *Male* | 85 | 72 (85%) | 13 (15%) |  |
| *Female* | 39 | 38 (97%) | 1 (3%) |  |
| Donor/Recipient Gender Match |  |  |  | 0.162 |
| *No* | 66 | 58 (88%) | 8 (12%) |  |
| *Yes* | 58 | 52 (90%) | 6 (10%) |  |
| Recipient Height (z-Score) | 126 | -1.40 ± 1.53 | -1.84 ± 2.03 | 0.501 |
| Recipient Weight (z-Score) | 126 | -1.27 ± 1.47 | -1.99 ± 1.86 | 0.309 |
| Recipient BMI (z-Score) | 126 | -0.54 ± 1.75 | -1.07 ± 2.12 | 0.354 |
| Diagnosis |  |  |  | 0.359 |
| *BA/Cholestasis* | 64 | 59 (92%) | 5 (8%) |  |
| *Metabolic* | 22 | 21 (95%) | 1 (5%) |  |
| *ALF* | 17 | 13 (76%) | 4 (24%) |  |
| *Others* | 23 | 19 (83%) | 4 (17%) |  |
| Recipient CMV Serostatus |  |  |  | 0.653 |
| *Negative* | 91 | 82 (90%) | 9 (10%) |  |
| *Positive* | 33 | 29 (88%) | 4 (12%) |  |
| Donor CMV Serostatus |  |  |  | 0.878 |
| *Negative* | 76 | 66 (87%) | 10 (13%) |  |
| *Positive* | 47 | 44 (94%) | 3 (6%) |  |
| Blood Group Mismatch |  |  |  | 0.339* |
| *No* | 106 | 94 (89%) | 12 (11%) |  |
| *Minor* | 15 | 15 (100%) | 0 (0%) |  |
| *Major* | 3 | 1 (33%) | 2 (67%) |  |
| Graft Type |  |  |  | 0.088 |
| *Whole* | 18 | 16 (89%) | 2 (11%) |  |
| *Split* | 68 | 61 (90%) | 7 (10%) |  |
| *Reduced* | 40 | 35 (88%) | 5 (13%) |  |
| CIT (Minutes) | 113 | 593 ± 121 | 559 ± 115 | 0.293 |
| Biliary Complications | 126 |  |  | 0.805 |
| *No* |  | 100 (88%) | 13 (12%) |  |
| *Yes* |  | 12 (92%) | 1 (8%) |  |
| Vascular Complication | 126 |  |  | 0.888 |
| *No* |  | 100 (88%) | 13 (12%) |  |
| *Yes* |  | 12 (92%) | 1 (8%) |  |
| Any Acute TCMR | 126 |  |  | 0.944 |
| *No* |  | 51 (89%) | 6 (11%) |  |
| *Yes* |  | 61 (88%) | 8 (12%) |  |
| Autoantibodies (Five Years) | 122 |  |  | 0.085 |
| *Negative* |  | 91 (90%) | 10 (10%) |  |
| *Positive* |  | 18 (86%) | 3 (14%) |  |
| Autoantibodies (Ten Years) | 97 |  |  | 0.668 |
| *Negative* |  | 83 (90%) | 9 (10%) |  |
| *Positive* |  | 4 (80%) | 1 (20%) |  |

*Continuous factors are reported as median (interquartile range) or mean ± SD, with p-values from Spearman’s correlation coefficients. Nominal factors are reported as N (Row %), with p-values from Mann-Whitney U or Kruskal-Wallis tests, unless stated otherwise. All p-values were generated using all four categories of the inflammation/fibrosis scores, which were subsequently combined to none/mild and moderate/severe for reporting in the table for brevity. Bold p-values are significant at p<0.05. *p-Value from Spearman’s correlation coefficient, as the factor is ordinal. acute TCMR, acute T-cell mediated rejection; ALF, acute liver failure; BA, biliary atresia; BMI, body mass index; CMV, cytomegalovirus; CIT, cold ischemic time; Pred, prednisolone; Tac, tacrolimus.*

**Supplementary Table G - Associations with fibrosis at ten years in the Tac+Pred cohort.**

|  | **Ten Year Fibrosis** | | | |
| --- | --- | --- | --- | --- |
|  | ***N*** | ***None/Mild*** | ***Moderate/Severe*** | ***p-Value*** |
| Recipient Age (Years) | 65 | 1.4 (0.8-3.9) | 1.5 (0.7-2.7) | 0.529 |
| Donor Age (Years) | 64 | 18.0 (11.7-27.8) | 21.3 (13.6-48.2) | 0.068 |
| Recipient Gender |  |  |  | 0.592 |
| *Male* | 39 | 28 (72%) | 11 (28%) |  |
| *Female* | 26 | 21 (81%) | 5 (19%) |  |
| Donor Gender |  |  |  | 0.823 |
| *Male* | 40 | 30 (75%) | 10 (25%) |  |
| *Female* | 22 | 17 (77%) | 5 (23%) |  |
| Donor/Recipient Gender Match |  |  |  | 0.699 |
| *No* | 30 | 24 (80%) | 6 (20%) |  |
| *Yes* | 32 | 23 (72%) | 9 (28%) |  |
| Recipient Height (z-Score) | 65 | -1.68 ± 1.63 | -1.54 ± 1.51 | 0.698 |
| Recipient Weight (z-Score) | 65 | -1.58 ± 1.54 | -0.96 ± 1.19 | 0.838 |
| Recipient BMI (z-Score) | 65 | -0.64 ± 1.83 | 0.19 ± 1.57 | 0.195 |
| Diagnosis |  |  |  | 0.882 |
| *BA/Cholestasis* | 40 | 31 (78%) | 9 (23%) |  |
| *Metabolic* | 10 | 7 (70%) | 3 (30%) |  |
| *ALF* | 5 | 3 (60%) | 2 (40%) |  |
| *Others* | 10 | 8 (80%) | 2 (20%) |  |
| Recipient CMV Serostatus |  |  |  | 0.269 |
| *Negative* | 47 | 35 (74%) | 12 (26%) |  |
| *Positive* | 18 | 14 (78%) | 4 (22%) |  |
| Donor CMV Serostatus |  |  |  | 0.139 |
| *Negative* | 45 | 34 (76%) | 11 (24%) |  |
| *Positive* | 20 | 15 (75%) | 5 (25%) |  |
| Blood Group Mismatch |  |  |  | 0.520* |
| *No* | 53 | 39 (74%) | 14 (26%) |  |
| *Minor* | 9 | 9 (100%) | 0 (0%) |  |
| *Major* | 0 | 0 (0%) | 0 (0%) |  |
| Graft Type |  |  |  | 0.575 |
| *Whole* | 12 | 10 (83%) | 2 (17%) |  |
| *Split* | 29 | 23 (79%) | 6 (21%) |  |
| *Reduced* | 24 | 16 (67%) | 8 (33%) |  |
| CIT (Minutes) | 59 | 614 ± 104 | 552 ± 157 | 0.623 |
| Biliary Complications | 65 |  |  | 0.734 |
| *No* |  | 45 (74%) | 16 (26%) |  |
| *Yes* |  | 4 (100%) | 0 (0%) |  |
| Vascular Complication | 65 |  |  | 0.280 |
| *No* |  | 44 (77%) | 13 (23%) |  |
| *Yes* |  | 5 (63%) | 3 (38%) |  |
| Any Acute TCMR | 65 |  |  | 0.493 |
| *No* |  | 19 (68%) | 9 (32%) |  |
| *Yes* |  | 30 (81%) | 7 (19%) |  |
| Autoantibodies (Five Years) | 64 |  |  | 0.669 |
| *Negative* |  | 34 (74%) | 12 (26%) |  |
| *Positive* |  | 14 (78%) | 4 (22%) |  |
| Autoantibodies (Ten Years) | 60 |  |  | 0.705 |
| *Negative* |  | 41 (72%) | 16 (28%) |  |
| *Positive* |  | 3 (100%) | 0 (0%) |  |

*Continuous factors are reported as median (interquartile range) or mean ± SD, with p-values from Spearman’s correlation coefficients. Nominal factors are reported as N (Row %), with p-values from Mann-Whitney U or Kruskal-Wallis tests, unless stated otherwise. All p-values were generated using all four categories of the inflammation/fibrosis scores, which were subsequently combined to none/mild and moderate/severe for reporting in the table for brevity. Bold p-values are significant at p<0.05. *p-Value from Spearman’s correlation coefficient, as the factor is ordinal. acute TCMR, acute T-cell mediated rejection; ALF, acute liver failure; BA, biliary atresia; BMI, body mass index; CMV, cytomegalovirus; CIT, cold ischemic time; Pred, prednisolone; Tac, tacrolimus*

**Supplementary Table H - Intention-to-treat analysis of ten-year protocol biopsies**

|  | **CSA-Only (+ N=12 Pred Restarted)** | **Pred+Tac** | **p-Value** |
| --- | --- | --- | --- |
| Histology Findings |  |  | **<0.001** |
| *Near Normal* | 24 (34%) | 48 (74%) |  |
| *Chronic Hepatitis* | 42 (60%) | 10 (15%) |  |
| *Others* | 4 (6%) | 7 (11%) |  |
| Inflammation |  |  | **<0.001** |
| *Normal* | 13 (19%) | 41 (63%) |  |
| *Mild* | 45 (65%) | 20 (31%) |  |
| *Moderate* | 11 (16%) | 4 (6%) |  |
| *Severe* | 0 (0%) | 0 (0%) |  |
| Fibrosis |  |  | 0.187 |
| *Normal* | 16 (24%) | 19 (29%) |  |
| *Mild* | 28 (42%) | 30 (46%) |  |
| *Moderate* | 15 (23%) | 16 (25%) |  |
| *Severe* | 7 (11%) | 0 (0%) |  |

*In this analysis, the CSA-Only group additionally includes biopsies from 12/14 of the patients who were excluded from the primary analysis, due to restarting prednisolone prior to the 10-year biopsy. These patients had biopsies performed approximately five years after restarting prednisolone; hence, the N=12 additional biopsies included in the analysis were a mean of 13 years (range: 10-17) post-transplant. The remaining two patients who restarted prednisolone were excluded, since no biopsy data were available. Data were also unavailable for the two patients in the Pred+Tac group who stopped prednisolone prior to ten years; hence, these were not included in the analysis. Data are reported as N (Column %), with p-values from Fisher's exact test for the histology findings, and Mann-Whitney U tests for inflammation/fibrosis; bold p-values are significant at p<0.05. CSA, cyclosporine A; Pred, prednisolone; Tac, tacrolimus.*
